# Evaluating Probenecid or Oseltamivir Inhibition of Influenza A Virus Replication Through Plaque Assay or Fluorescent Focus Assay Using Non-Structural Protein 1–H1N1 Venus Reporter Virus

**DOI:** 10.3390/v17030335

**Published:** 2025-02-27

**Authors:** Jackelyn Murray, Aitor Nogales, Luis Martinez-Sobrido, David E. Martin, Fred D. Sancilio, Ralph A. Tripp

**Affiliations:** 1 Department of Infectious Diseases, University of Georgia, Athens, GA 30602, USA; jcrab@uga.edu; 2 Center for Animal Health Research, CISA-INIA-CSIC, 28130 Madrid, Spain; nogales.aitor@inia.csic.es; 3 Texas Biomedical Research Institute, San Antonio, TX 78227, USA; lmartinez@txbiomed.org; 4 TrippBio, Inc., Jacksonville, FL 32256, USA; davidmartin@trippbio.com; 5 Department of Chemistry and Biochemistry, Florida Atlantic University, Jupiter, FL 33431, USA; fredsancilio@clearwayglobal.com

**Keywords:** influenza virus, probenecid, oseltamivir, NHBE cells, antiviral, plaque assay, fluorescent focus assay

## Abstract

It is essential to understand the molecular mechanisms of influenza antiviral therapeutics to evaluate their efficacy. Virus plaque assays are commonly used to assess the antiviral effects of drugs on virus replication; however, this method is labor-intensive and can present challenges. We avoided this method by using a replication-competent influenza A virus (IAV) expressing a reporter fluorescent gene fused to the non-structural protein 1 (NS1) gene. The reporter IAV was detectable in normal human bronchoepithelial (NHBE) infected cells and offered an improved method to determine the therapeutic efficacy of the antiviral drugs probenecid and oseltamivir compared to a standard plaque assay. This method provides an excellent means for evaluating therapeutic approaches against IAV.

## 1. Introduction

Plaque assays are one of the most accurate methods for directly quantifying infectious replicating viruses. The main limitation of a plaque assay is that it can only be used with viruses that form visible plaques by killing host cells. The assay involves adding viruses to permissive cells and applying a semi-solid overlay to limit the spread of infection to neighboring cells. Cell death leads to the formation of plaques. Plaque assays require that the cultured cells to be assessed are susceptible to the virus. Typically, the virus is diluted several times, and each dilution is added to the cells and then incubated to allow the virus to attach to target cells. Subsequently, the cells are covered with a gel or semisolid overlay. The replicating viruses release progeny virus, and the gel restricts particle movement so that newly produced viruses can only infect neighboring cells. The virus-infected cells die and create a hole in the cell monolayer, forming a plaque. The cells are then fixed and stained with a dye for contrast, making counting plaques easier to visualize. Cells that remain adhered to are assumed to be uninfected, while plaques are considered to arise from cell death caused by infection. This process allows one to determine the viral titer, measured as plaque-forming units/mL (PFU/mL). There are caveats when interpreting the results of a plaque assay. Specifically, one cannot know if there is a one-to-one ratio of plaques to infectious viruses, and the virus titer determined is specific to the plaque assay conditions, as virus infectivity is affected by numerous factors, including the type of host cell and virus input.

Plaque assay principles, such as focus-forming assays (FFAs), can also be used. FFAs do not rely on cell lysis and counterstaining to detect plaque formation but instead employ immunostaining to detect viral proteins through antibodies directly. FFAs have the advantage of increased sensitivity, decreased incubation times after infection, and, most importantly, the ability to quantify non-lytic viruses. We exploited an FFA to screen and evaluate antiviral drug sensitivity using a replication-competent influenza A/Puerto Rico/8/1934 H1N1 (PR8) virus to avoid issues associated with traditional plaque assays. One potential problem is that influenza infection of A549 cells is very sensitive to trypsin. Thus, a more relevant human cell model, such as normal human bronchoepithelial (NHBE) cells, is used.

In this assay, the fluorescent green transgene (Venus) was fused to the influenza non-structural protein 1 (NS1), allowing for the direct measurement of the impact of an antiviral drug on virus replication. Previously, this PR8 NS1–Venus chimeric protein was shown to be stable and bright, and the chimeric virus does not need to be plaque-purified for its application [1]. Additionally, it was demonstrated that replication-competent recombinant PR8 virus with the NS1 substituted with a reporter, e.g., fluorescent or luciferase proteins, can be used to directly compare the results from a standard plaque to an FFA using rIAV with the NS1 substituted with a Venus green reporter gene on antiviral efficacy.

There are two categories of antiviral drugs: direct-acting antivirals (DAAs) and host-directed antivirals (HDAs) [2]. DAAs target the virus, while HDAs target host factors or cell pathways required for virus replication. Typical influenza antivirals are M2 proton channel antagonists (amantadine), rimantadine, neuraminidase inhibitors (NAIs, including zanamivir, oseltamivir, peramivir, and laninamivir), and polymerase acidic endonuclease inhibitors (baloxavir marboxil) [3,4,5]. Probenecid, a uricosuric agent approved in 1951 to treat gout, has broad-spectrum antiviral activity against several respiratory viruses; it can inhibit H5N1 replication, and it can reduce serum pro-inflammatory cytokine expressed in response to infection [6]. We compared the antiviral effectiveness of the neuraminidase inhibitor, oseltamivir, against probenecid. Oseltamivir is a prodrug of oseltamivir carboxylate and a selective inhibitor of neuraminidase. The FDA approved oseltamivir (Tamiflu™, F. Hoffmann-La Roche Ltd., Basel, Switzerland) as an oral treatment for uncomplicated influenza [7]. We tested the therapeutic performance of these antiviral drugs and whether probenecid, which targets the host cells, has superior antiviral efficacy compared to oseltamivir. We show that the FFA results are comparable to the plaque assay and that they can be determined more rapidly, with good sensitivity, to study the replication of IAV without performing a plaque assay [8].

## 2. Materials and Methods

### 2.1. Cell Culture

Primary normal human bronchial epithelial (NHBE) cells (Lonza, Basel, Switzerland; CC-2540) were grown in Bronchial Epithelial Cell Growth Medium (BEGM, Lonza, CC-3170). The cells were maintained in the log phase at 37 °C in a 5% CO_2_ incubator and used for probenecid studies. Madin–Darby canine kidney (MDCK) cells (ATCC CCL-34) were maintained in Dulbecco’s Modified Eagle Medium (DMEM), Thermofisher (Waltham, MA, USA) supplemented with 5% fetal bovine serum (Sigma, St. Louis, MO, USA) and grown at 37 °C with 5% CO_2_.

### 2.2. Antiviral Drugs

Probenecid (Sigma, CAS Number: 57-66-9) was diluted in DMSO (Sigma) and resuspended in media to make the desired concentrations. Oseltamivir carboxylate (Sigma, CAS Number: 204255-11-8), the active metabolite of oseltamivir phosphate (Tamiflu^®^), was diluted in water.

### 2.3. Influenza A/Puerto Rico/8/1934 H1N1 Venus Reporter Virus (PR8-Venus)

Influenza A/Puerto Rico/08/1934 H1N1 (PR8) expressing Venus (PR8-Venus) was generated as previously described for a PR8 expressing mCherry [9]. The viral stock was made by infecting (multiplicity of infection (MOI) = 0.1) MDCK cells and incubating them in the presence of tosyl phenylalanyl chloromethyl ketone (TPCK)-trypsin (Sigma) for 72 h at 37 °C and 5% CO_2_. A plaque assay using MDCK cells was used to quantify the titer.

### 2.4. Influenza Plaque Assay

Ten-fold serial dilutions of cell supernatants were added to confluent MDCK cell monolayers in 12-well plates supplemented with 1 μg/mL of TPCK trypsin. Following a 1 h virus adsorption at 37 °C, 5% CO_2_, 2 mL of overlay containing 1-part medium consisting of 10× MEM supplemented with 200 mM L-glutamine (Gibco/ThermoFisher), HEPES solution (Gibco/ThermoFisher), 7.5% NaCHO_3_ (Gibco/ThermoFisher), and 1-part 2.4% Avicel (FMC BioPolymer, Philadelphia, PA) in water and 1 μg/mL of TPCK-trypsin was added/well. Samples were incubated at 37 °C, 5% CO_2_ for 3 days, washed in PBS, and fixed with 60:40 acetone: methanol (Sigma) for 20 min at room temperature. Cells were counterstained with crystal violet, and plaques were counted.

### 2.5. Probenecid or Oseltamivir Treatment to Determine Influenza Replication Using a Plaque Assay or a Fluorescent Focus Assay

Probenecid (Sigma, St. Louis, MO, USA) dilutions were made from a 100 mM working stock. For prophylactic or therapeutic treatment studies, NHBE cells were plated overnight at 2.5 × 10^4^ cells/well in 96-well flat-bottom plates (Costar/ThermoFisher). Cells were pretreated prophylactically for 24 h before infection or therapeutically treated at 1 hpi with probenecid or oseltamivir at different concentrations, i.e., 100, 50, 25, 12, 6, 3, 1, 0.5, 0.2, 0.1, and 0 µM. NHBE cells were plated in 24-well plates (Corning, Corning, NY, USA). For the prophylactically treated cells, the tissue culture media were decanted, the cells were washed with PBS (Gibco/Thermofisher), and probenecid or oseltamivir diluted in BEGM media was added to the wells and incubated for 24 h. For FFAs, PR8-Venus at MOI = 0.1 in infection media containing 1 μg/mL of TPCK-trypsin was added to the cells for prophylactic and therapeutic treatment groups. After removing the reporter virus, the infection was incubated for 1 h at 37 °C. Probenecid or oseltamivir was added to the respective wells in BEGM media containing 1 μg/mL of TPCK-trypsin, and the assay continued at 37 °C for 48 h. Supernatants were removed and used for plaque assays, and fresh media containing 1 μg/mL of Hoechst dye (Thermofisher) were added to the wells to enable nuclear detection. The plates were imaged on an ArrayScan (Thermofisher). A loss of fluorescent signal after drug treatment indicated inhibition of virus replication.

### 2.6. Statistics

GraphPad Prism 9 (GraphPad Software, Boston, MA, USA) was used to calculate all of the IC_50_s using a nonlinear regression model based on the Hill equation.

## 3. Results

This study examined oseltamivir, which can prevent IAV and IBV replication [10]. We compared probenecid’s antiviral efficacy to oseltamivir’s to inhibit IAV replication, as we previously showed robust antiviral activity of probenecid and oseltamivir on influenza virus replication [11]. To evaluate antiviral drug effectiveness without a traditional plaque assay, we examined FFAs using a PR8-Venus reporter virus [9] and compared probenecid to oseltamivir treatment. The level of virus replication was determined by quantifying Venus reporter expression levels. NHBE cells were prophylactically treated with probenecid or oseltamivir for 24 h before infection or treated 1 h after infection (i.e., 100–0 µM). At 48 hpi, live cells were stained using Hoechst dye to enable nuclear detection, and the wells were imaged using an ArrayScan (Thermofisher) high-content imager. The results showed that probenecid treatment was superior to oseltamivir for inhibiting virus replication. The 50% inhibitory concentration (IC_50_) of probenecid was 0.07 µM for prophylactically treated cells and 0.02 µM for therapeutically treated cells. The IC_50_ for oseltamivir was 0.29 µM for prophylactically treated cells and 1 µM for therapeutically treated cells (Table 1).

**Figure 1:**
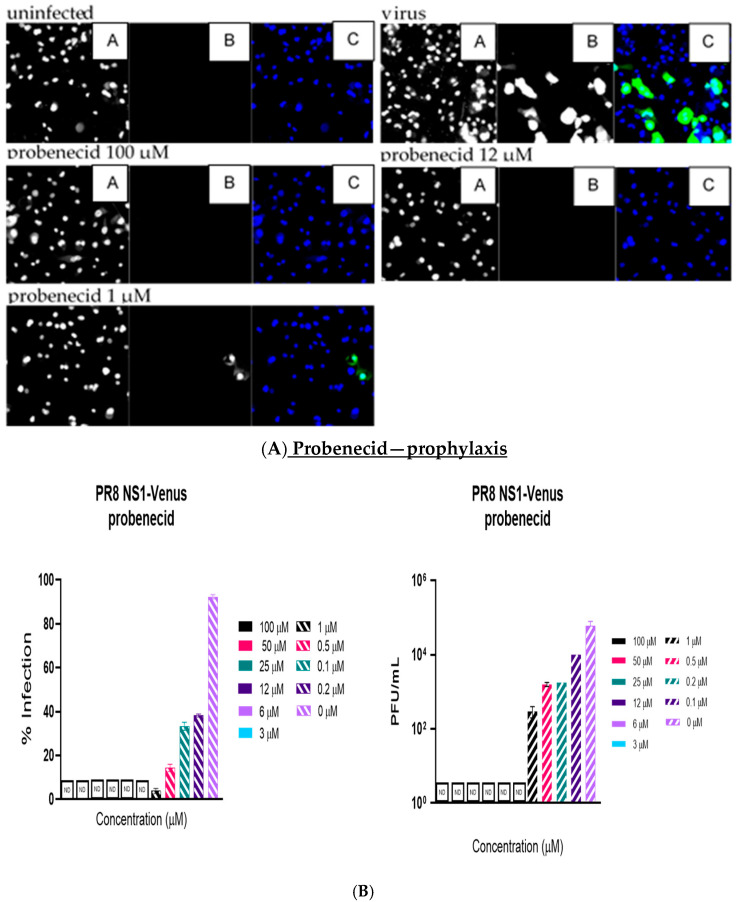
NHBE cells were prophylactically treated with probenecid for 24 h before infection with PR8 NS1-Venus (MOI = 0.1). Infection proceeded for 48 h, after which the cells were analyzed. (**A**) FFAs were performed on an ArrayScan. Panel A is nuclei (Hoechst dye staining), panel B is IAV infection, and panel C is the composite image of the two. (**B**) Supernatants were collected and titrated on MDCK cells to perform traditional plaque assays. Statistical analysis was performed via one-way ANOVA. The panels are 20×, as determined using the ArrayScan (Thermofisher). There was no detectable virus (ND) in the 3–100 µM concentrations, indicated by ND.

**Figure 2:**
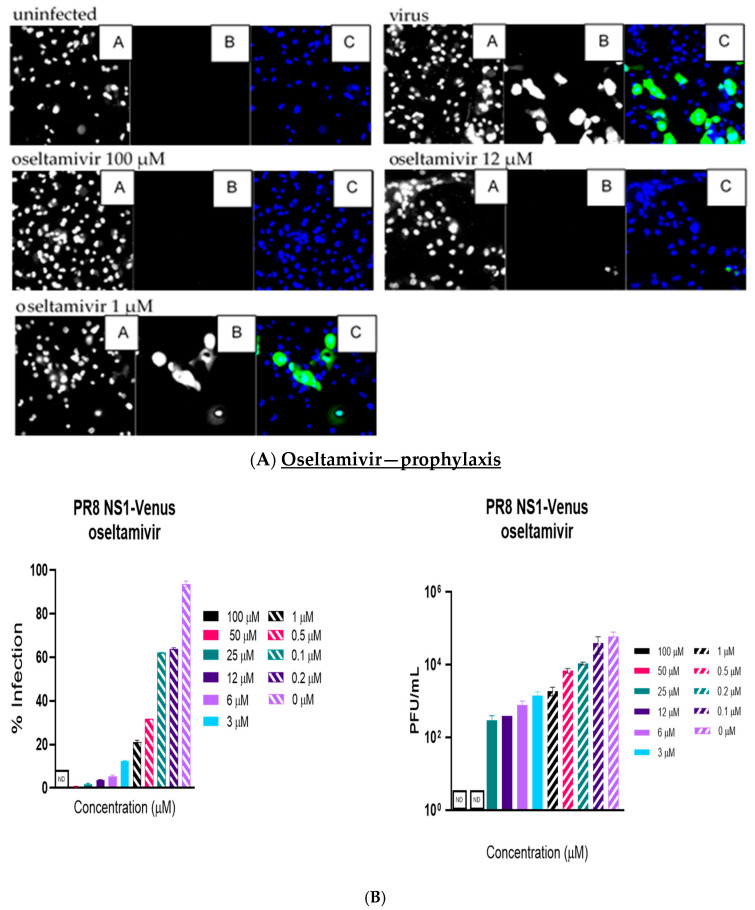
NHBE cells were prophylactically treated with oseltamivir for 24 h before infection with PR8 NS1-Venus (MOI = 0.1). Infection proceeded for 48 h, after which the cells were analyzed. (**A**) FFAs were performed on an ArrayScan. Panel A is nuclei (Hoechst dye staining), panel B is IAV infection, and panel C is the composite image of the two. (**B**) Supernatants were collected and titrated on MDCK cells to perform traditional plaque assays. Statistical analysis was performed via one-way ANOVA. Statistical analysis was performed via one-way ANOVA. The panels are 20×, as determined using the ArrayScan. There was no detectable (ND) virus in the 100 µM concentration for the FFA and ND virus in the 50–100 µM concentrations.

**Figure 3:**
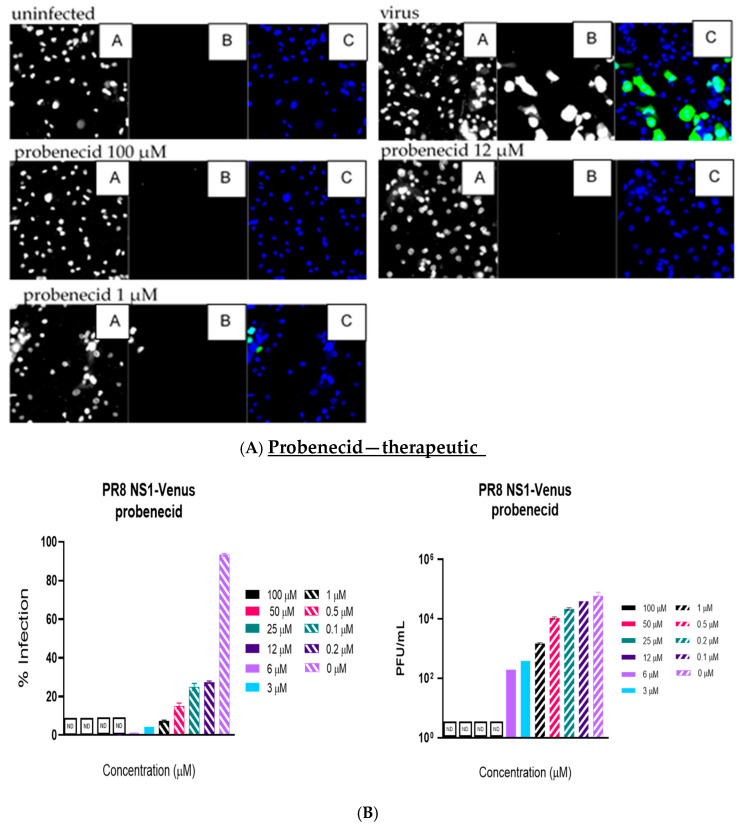
NHBE cells were infected for 1 h with PR8 NS1-Venus (MOI = 0.1) and therapeutically treated with probenecid. Infection proceeded for 48 h, and the cells were analyzed. (**A**) FFAs were performed on an ArrayScan. Panel A is nuclei (Hoechst dye staining), panel B is IAV infection, and panel C is the composite image of the two. (**B**) Supernatants were collected and titrated on MDCK cells to perform traditional plaque assays. Statistical analysis was performed via one-way ANOVA. No detectable (ND) virus was detected in the 12–100 µM concentrations.

**Figure 4:**
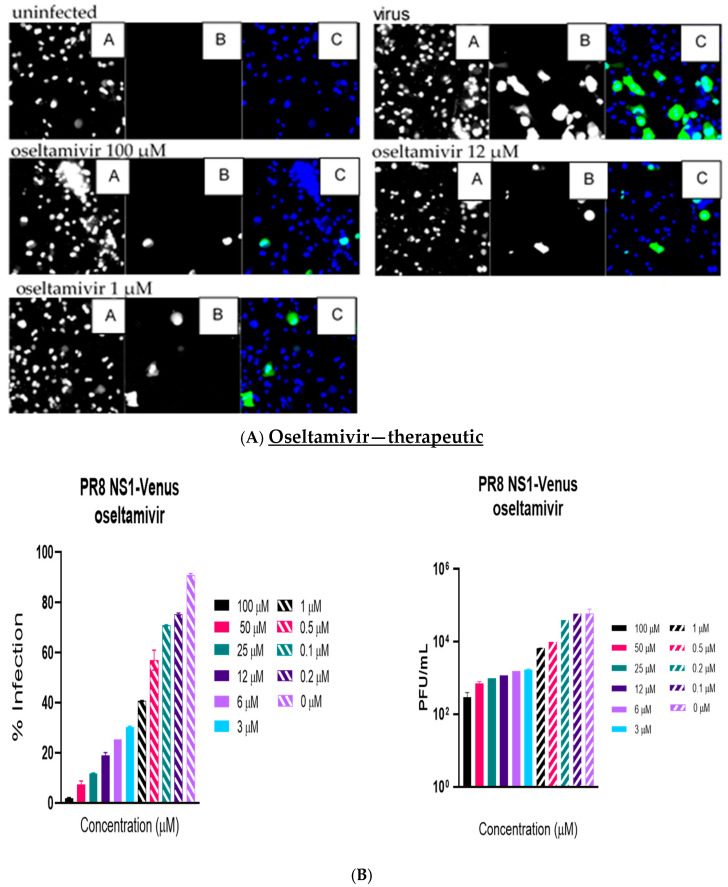
NHBE cells were infected for 1 h with PR8 NS1-Venus (MOI = 0.1) and therapeutically treated with oseltamivir. Infection proceeded for 48 h, and the cells were analyzed. (**A**) FFAs were performed on an ArrayScan. Panel A is nuclei (Hoechst dye staining), panel B is IAV infection, and panel C is the composite image of the two. (**B**) Supernatants were collected and titrated on MDCK cells to perform traditional plaque assays. Statistical analysis was performed via one-way ANOVA.

Like the plaque assay results, probenecid and oseltamivir prophylactic and treatment were evaluated for inhibition of PR8-Venus replication in NHBE cells treated with 100, 50, 25, 12, 6, 3, 1, 0.5, 0.2, 0.1, and 0 µM concentrations. Drug efficacy was apparent based on the absence of Venus fluorescence in the cells, which indicated a lack of virus replication. All drugs and dilutions were imaged in two independent FFAs, and all drug dilutions were examined in triplicate. Only 100, 12, or 1 μM responses are shown for probenecid or oseltamivir (Figure 1, Figure 2, Figure 3 and Figure 4) treatment for brevity. No Venus fluorescence was detected in NHBE cells treated with 100, 50, 25, 12, 6, or 3 µM concentrations of probenecid. In contrast, oseltamivir prophylactic treatment inhibited PR8-Venus replication in NHBE cells treated at 100, 50, 25, 12, 6, or 3 µM concentrations. These results show that drug efficacy can be screened by evaluating PR8-Venus replication in NHBE cells, which is more efficient and does not require the manipulation and level of virology needed for plaque assays.

## 4. Discussion

We have previously shown that probenecid is safe and effective in limiting IAV replication [6,11,12]. We showed that IAV replication is inhibited by probenecid treatment in A549 cells and that probenecid had similar IC_50_ values for other isolates, including A/HKx31 (x31) (IC_50_ = 0.03 μM), A/Viet Nam/1203/2004(H5N1) (IC_50_ = 0.03 μM), and A/Anhui/1/2013 (H7N9) (IC_50_ = 0.016 μM). Notably, probenecid was more potent than oseltamivir for these IAVs when given as a prophylactic or therapeutic treatment. We also examined the effects of probenecid or oseltamivir on oseltamivir-sensitive (A/Mississippi/3/2001 H1N1) and oseltamivir-resistant (A/Mississippi/3/2001 H1N1 H275Y) infected A549 cell lines [11]. The IC_50_ for prophylactic treatment with probenecid was 0.0002 μM, and it was 0.0014 μM for oseltamivir treatment. The IC_50_ for drug treatment 1 h after infection with probenecid was 0.001 μM, and it was 0.045 μM for oseltamivir treatment of A/Mississippi/3/2001 H1N1-infected cells. To determine the effect of drug resistance on the antiviral activity profile, A549 cells were infected with oseltamivir-resistant A/Mississippi/3/2001 H1N1 H275Y. The IC_50_ for probenecid was 0.0002 μM, and it was 0.013 μM for oseltamivir. The IC_50_ for probenecid treatment 1 h after infection was 0.0009 μM, and it was 0.049 μM for oseltamivir. The results showed that probenecid reduced IAV replication better than oseltamivir for both treatments, and the results were emulated in the FFA.

In this study, we determined the IC_50_ based on the percentage of infection observed in the FFA, followed by the percentage of infection determined from the PFU/mL in the plaque assays using MDCK cells. This approach was taken because NHBE cells do not form plaques but represent a physiologically relevant model. Also, A549 cells are from a single type of clone from alveolar basal epithelial cells. In contrast, NHBE cells are isolated from the epithelial lining of airways above the bifurcation of the lungs. Differences between the source tissue and the differentiated primary cells may impact infection studies [13] and may have contributed to IC_50_ differences. FFAs do not rely on cell lysis and counterstaining to detect plaque formation; instead, using an NS1-H1N1 Venus reporter virus, direct detection of replication can be performed. This offers increased sensitivity of virus detection and decreased incubation times after infection. Also, as previously indicated, NHBE cell supernatants were titrated on MDCK cells for the traditional plaque assays with a detection limit of approximately 10 PFUs, whereas PR8 NS1-Venus (MOI = 0.1) is detectable upon virus replication.

IAV encodes the NS1 protein, which is highly expressed during viral infection [14]. NS1 is a multifunctional protein and virulence factor that contributes to viral replication by suppressing interferon (IFN) production and the antiviral activity of many IFN-stimulated genes (ISGs) [15]. Recombinant IAVs expressing reporter genes from the NS segment have been shown to eliminate the need for approaches to determine the presence of the virus, and these recombinant IAVs have been shown to resist the loss of reporter gene expression after viral passaging [9]. We show that FFAs using rIAV expressing the Venus green reporter gene can be applied as a useful screening tool for antiviral medications, and the results of FFA screens can yield more rapid results with similar sensitivity to traditional plaque assay results.

## Figures and Tables

**Table 1:** Inhibition of influenza virus replication by chemotherapy drugs.

Prophylactic Scheme	Schedule/Regimen	Therapeutic Scheme
	IC_50_	IC_50_
Probenecid	0.07 µM	0.02 µM
Oseltamivir	0.29 µM	1 µM

## Data Availability

The data supporting the reported results are in the Tripp laboratory at the University of Georgia.
